# Midpoint Relay Selection Using Social Trust and Battery Level to Enhance Throughput in Cooperative Device-to-Device Communications

**DOI:** 10.3390/s20216007

**Published:** 2020-10-23

**Authors:** Ushik Shrestha Khwakhali, Prapun Suksompong, Steven Gordon

**Affiliations:** 1 School of Information, Computer and Communication Technology, Sirindhorn International Institute of Technology, Thammasat University, Pathum Thani 12120, Thailand; d5722300372@g.siit.tu.ac.th; 2 School of Engineering and Technology, CQUniversity, Cairns, QLD 4870, Australia; s.d.gordon@cqu.edu.au

**Keywords:** cooperative networks, device-to-device (D2D) communications, social-aware relay selection, social trust, battery level, relay transmission power, throughput

## Abstract

Device-to-device communications in underlay mode has emerged as a promising way to enhance spectrum efficiency in cellular networks. Recently, relay selection in D2D communications underlaying cellular networks is gaining more research interest. In this paper, we propose two relay selection schemes for D2D communications underlaying cellular networks, Midpoint Relay Selection using Social Trust and Battery Level (MRS-ST-BL) and Midpoint Relay Selection using Social Distance and Battery Level (MRS-SD-BL). These proposed schemes utilize battery power level information of devices together with social trust information of users in the network for relay selection. For performance evaluation, initially we show that the throughput of state-of-the-art schemes Hybrid Relay Selection (HRS) and our previously proposed schemes Midpoint Relay Selection using Social Trust (MRS-ST) and Midpoint Relay Selection Using Social Distance (MRS-SD) decrease, when relays have varying battery power. Then, we compare the performance of our proposed schemes against existing schemes including HRS, MRS-ST and MRS-SD. The performance comparison is done at various social trust scenarios and device densities. We show that our proposed schemes can significantly improve the throughput of D2D communications, particularly when relays have different battery power levels in weak social trust scenarios. Finally, we show that the performance of our proposed scheme MRS-ST-BL varies with the change in battery power threshold.

## 1. Introduction

### 1.1. D2D Communications

Unlike traditional mobile communications in which mobile devices communicate with each other via a base station (BS), device-to-device (D2D) communications allow mobile devices in proximity to directly communicate, bypassing the BS [1]. Increase in spectrum utilization, enhancement in transmission rate, data offloading, reduction in transmission delay, saving of power usage to improve network capacity are some of the major benefits of D2D communications [2,3]. D2D communications in underlay mode use the same frequency resources as that allocated for cellular communications as opposed to D2D communications in overlay mode which use dedicated cellular frequency resources [1]. D2D in underlay mode is one of the most promising ways to handle the shortage of frequency resources [2,4]. Recently research interest has increased to use a relay for D2D communications in underlay mode [5,6]. The focus of this paper is also to find a relay that maximizes the throughput of D2D communications in underlay mode, utilizing social information of users and battery power information of devices.

In this paper, we present the design of two midpoint relay selection schemes for D2D communication which utilizes social trust information of users and battery power of devices for relay selection. The design of two proposed schemes varies in the way they filter devices to select as a relay. We show that, when D2D communications using relays have different battery power levels, our proposed relay selection schemes significantly increase throughput.

### 1.2. Problems in Relay Selection

Generally, a relay is used to enhance performance of cooperative D2D communication networks. The selection of a relay that maximizes throughput of D2D communications become difficult when transmission power of the relay device is variable that is, when the relay is capable of choosing or adjusting its transmission power. This is because the signal-to-noise-ratio (SNR) of D2D communication depends upon the length of links from the source to the relay and that from the relay to the destination as well as transmission power of the relay. Recent studies [5,7,8] have put forward the idea of utilizing social information of users in the network for relay selection.

In our previous relay selection schemes [9,10,11] and other state-of-the-art schemes [7,12,13], it is assumed that a relay device always transmits at a power proportional to the social trust it has for the source. However, mobile devices being power constrained devices, may not always have full battery power that is, the battery power on mobile devices may vary at different times. When the battery power on a relay is low, the transmission power of the relay may not be proportional to the social trust. This makes the relay selection more challenging. Even in co-operative networks, it is possible that a relay having a low battery power may preserve its battery power by using a low transmit power while relaying data for other devices. In such scenarios, throughput of existing relay selection schemes may vary as compared to their performance when relay devices have full battery power. It is important to analyze how the transmission behaviour of a relay with varying battery power impacts the throughput of relay selection schemes in D2D communications. Additionally, designing a relay selection scheme that enhances throughput in such network scenario is necessary.

### 1.3. Related Work

This section summarizes recent work in the field of D2D communications utilizing social information of users. The survey papers, References [14,15], review the current advancements in D2D communications and how social information can be used in the design of D2D systems. Social strength estimation techniques are proposed in Reference [16,17]. Different energy efficient approaches for D2D communications are proposed in References [1,13,18,19]. Multi-hop D2D communications are used in References [20,21] and multicasting using D2D communications in Reference [22]. Social information of network users is used for relay selection to improve throughput of D2D communications in References [5,7,8,12] and in our previous papers [9,10,11]. These papers and issues are reviewed in the remainder of this section.

Kar et al. [14] presented a brief overview of the standardization of D2D communication based on 3GPP Release 12 and subsequent releases. They described the reference architecture for D2D communication, channel structure and features of D2D communications. Additionally, they discussed different challenges present in D2D communications and also highlighted some of the important areas of interaction between 5G and D2D communications. Nitti et al. [15] presented a survey on D2D communications focused on the utilization of social networks with the aim to explore and understand how the integration of social-awareness benefits D2D communications. Their survey identified social communities, social link/tie and social centrality as major social features of social network that can be used in D2D communications. Furthermore, they identified the key issues in D2D communications. The key issues are relay discovery and peer selection, communication mode selection, and spectrum resource allocation and management. Additionally, they summarized different proposed solutions using social networking.

Social-interactions are now considered as a novel and imperative dimension in the design of communication systems [23]. Social relationship information of users can be used in D2D communications to reduce load on cellular systems [4]. The social relationship information obtained from online social networks are more detailed and complex in structure as compared to offline social networks [24].

Bandford et al. [16] proposed a method to estimate social tie strength in a network that consists of small number of users. They found that for a network having less than hundred users, social tie strength can be estimated using aggregated call duration. Xiang et al. [17] developed a social strength estimation which is based on interaction information of network users (e.g., communication, tagging) and similarities in account profile of users. The continuous-valued social strength estimated from the model is capable to provide link weights with higher autocorrelation, leading to improved accuracy in social trust estimation. However, in this paper we use Pareto distribution to model the social trust among users. This approach is also used in other studies [6,12,19].

Generally, mobile devices have limited storage capacity and are power constrained. In relay assited D2D communication networks, relay devices which are unselfish might always forward data for others, and eventually run out of battery power. On the other hand, devices that are selfish might always use a relay to enhance their D2D communications, without forwarding data for others [5]. Therefore, it is necessary to design a D2D relay selection mechanism that motivates devices to relay data for others.

Datsika et al. [18] proposed energy efficient medium access protocol for cooperative D2D communications that utilizes social information of users. They highlight some of the practical issues when social information is used in D2D communications.

Rahman et al. [1] proposed energy efficient relay selection scheme for multi-relay D2D communications underlaying cellular systems. They used adaptive neuro-fuzzy inference system architecture for relay selection and a particle swarm optimization algorithm is used for power allocation with the aim to maximize network energy efficiency. However, social information is not utilized to select a relay.

Sun et al. [13] proposed social-trust and power-efficient relay selection (STRS) scheme for D2D communications underlaying distributed shared network, which takes into account physical and social information of users. In their scheme, initially candidate relay users are selected using location and social trust information. Then among the candidate relay devices, a device having lowest power consumption is selected as a relay.

Unlike References [1,13,18], we aim to maximize throughput by selecting a relay transmitting at a high power and minimize the energy consumption by limiting the number of probes for the selection of a relay.

Li et al. [19] proposed social-aware energy efficient relay selection (SERS) scheme for cooperative D2D communications. Social trust, encounter history and node energy consumption are utilized for the selection of a relay. They formulated power control game that maximizes data rate while minimizing energy consumption and interference. The energy consumption and network interference is minimized by using social distance as the penalty coefficient. Similar to Reference [19], we measure the average throughput that can be achieved by our proposed schemes.

Ying et al. [20] developed an algorithm to select relays for content dissemination in multi-hop D2D communications called PSRS which minimizes power consumption. They used frequency/duration of interaction for sharing/downloading of content and social reciprocity to quantify social relationship value.

Mishra et al. [21] proposed relay selection scheme for multi-hop D2D communication in which two devices are selected as candidate relays. The relay is selected by the BS and instantaneous SINR and neighbour discovery is done by D2D pair. When one of the devices is unable to relay, the second device is selected as a relay. The relays are chosen among the available devices which satisfy SINR threshold, battery power threshold, reliability and buffer requirements. Their scheme increases success probability and network throughput of D2D communications.

Chiti et al. [22] proposed multicasting in cooperative D2D communications using social-aware relay selection. Both the propagation link conditions and social-trust level are used while selecting a relay to minimize end-to-end content delivery delay.

While References [20,21,22] present schemes for D2D, none of them probes to know the physical condition of the path from the relay to the destination and do not consider the time/cost involved for probing during the selection of a relay.

The following papers are closely related to our work.

Chen et al. [8] developed a relay selection technique for cooperative D2D communications that leverages social trust among mobile users while selecting a relay. Social and physical distances are used for analysis of cooperative D2D communications. Their assumption is that a mobile device having high social trust for a source device relays data for the source at a high transmission power. When the mobile users do not have social relationship, relaying is done using social reciprocity. Their study shows that a relay selection based on social trust and/or social reciprocity can significantly improve average throughput of D2D communications. The improvement in the system increases with the increment in number of users. Their proposed mechanism can provide up to 122% performance gain over the case without D2D cooperation.

Li et al. [5] proposed an optimal stopping relay selection scheme to enhance throughput of D2D communications in a distributed network. The average contact duration and common friends are used to represent social relationship weight coefficient. The candidate relay nodes are filtered using social threshold. Then, a relay is selected using an optimal stopping approach. The proposed scheme significantly reduces the time taken for probing of devices before selection of a relay.

Similarly, Zhang et al. [7] proposed a decode and forward relaying for a cooperative D2D communications. The relay has a transmission power proportional to the social trust that is, the relay transmits at a high power when it has strong social relationship with the source. They used optimal stopping approach while probing socially trusted nodes sequentially to select a relay. This helps to maintain the balance between relay probing cost and performance gain. The major setback of their research is that the comparison of the proposed scheme is done with generic schemes (direct communication and random relay selection). Uniform distribution is used to assume the social trust among users, which is less realistic. Additionally, they analyzed the scheme only for a specific distance (100  m) between source and destination. However, in reality, source-destination distance can vary over time.

Pan and Wang [12] proposed a Hybrid Relay Selection (HRS) scheme in which social information of users are utilized for selection of a relay. We compare the performance of HRS scheme with relay selection schemes proposed in this paper. In HRS, mobile devices that have a strong relationship with each other are assumed to have high value of social trust between them. Such devices having strong social trust with a source can potentially relay data with high transmission power. Both link characteristics and social trust between users are considered for relay selection.

HRS selects a relay around the source. To select a relay, a source probes devices that are not engaged in communication and located within a circular region with the source at the center. Only those devices which have social trust greater than a threshold value are probed. HRS assumes the transmission power of a relay is linearly proportional to the social trust between the source and the relay. The social trust between the users in the network is modelled using Pareto distribution. The source calculates the data rate offered by each of the probed nodes and chooses the node that offers maximum data rate as a relay. The authors show that the average throughput of HRS is higher than two other schemes: Distance-based Relay Selection (DRS) and Social-based Relay Selection (SRS). They also suggested that the relay selection region should neither be too small nor too large. The major downside of HRS is that the throughput is low for small search radius due to frequent selection of long distance links. Also, the throughput is low for large search radius. This is because of the significant time duration spent for probing. Furthermore, the performance of HRS is not compared against relay selection schemes proposed by other researchers.

Our previous work [9] proposed a midpoint relay selection scheme that selects a socially trusted device located around the midpoint of the distance between the source and the destination. The scheme performs better than HRS. The drawback of the scheme is that it probes all the devices within the circular region having social trust above a threshold value. We overcame the limitation by proposing two relay selection schemes Midpoint Relay Selection Scheme using Social Trust and Midpoint Relay Selection Using Social Distance in Reference [10]. In Reference [11], we proposed a midpoint relay selection scheme that improves the throughput of D2D communications even when social trust among the users are strong, provided that the BS can accurately estimate social trust information of users.

Different from all the papers above, this paper proposes midpoint relay selection schemes that incorporates battery power of devices together with social trust information of users to select a relay with the aim to maximize average throughput of D2D communications.

### 1.4. Our Contributions

The key contributions of this research are as follows:

- We show that the throughput of existing state-of-the-art schemes (HRS [12], MRS-ST and MRS-SD [10]) reduce when relay devices have variable battery power levels.
- We propose new relay selection schemes MRS-ST-BL and MRS-SD-BL that optimize throughput of D2D communications using a socially trusted relay whenever possible. The optimization problem is formulated using social trust among users and battery power level on devices together with physical location of the devices. Social trust among users are used for filtering of devices for selection of a relay. Pareto distribution is used to model the social trust among users in the network. According to the pre-defined policy, relay devices adjust their transmission power depending upon social trust values for the source and battery power level on the devices to maximize the throughput.
- We evaluate the performance of our newly proposed relay selection schemes extensively through simulation by comparing with generic and state-of-the-art schemes. We show that our proposed schemes achieve higher throughput when devices in the network have varying battery power levels, in networks having different node densities and social trust scenarios. Additionally, we compare the average number of probes and transmission power of relay of our proposed schemes with various other schemes to show how our proposed schemes enhance throughput of D2D communications.

This paper is an extension of our previous papers midpoint relay selection schemes (MRS-ST and MRS-SD) [9,10]. We have significantly updated the design of relay selection by introducing battery power level of devices, which is more realistic. We have analyzed the performance of proposed schemes in various social trust scenarios and device densities by comparing with other schemes.

The rest of this paper is organized as follows. Section 2 introduces the D2D communication system model used in this paper. It includes the network model, mobility model, social trust model, battery model and communication link model. Section 3 presents details of our proposed schemes MRS-ST-BL and MRS-SD-BL. Section 4 explains the exchange of probing messages for selection of a relay in D2D communications. Section 5 presents the extensive analysis of our proposed schemes with other state-of-the-art schemes. Section 6 presents the conclusion of this paper.

## 2. D2D Communication System Model

### 2.1. Network Model

Consider a cooperative network which consists of $n_{total}$ mobile devices under the coverage of a BS. When a source (*s*) mobile device wants to have D2D communications with a destination (*d*) mobile device using a cellular channel, bypassing the BS, it can be done in two ways. The first way is to send data packets directly from the source to the destination. This is referred to as direct D2D communication. Another way is to initially send the data packets from the source to a relay (*r*) mobile device in proximity. The relay receives the packets and forwards them to the destination. This is referred to as D2D communication via a relay. Decode-and-forward (DF) relaying [7,8,12,25] is used for D2D communications to forward data. All mobile devices which are not engaged in communication are considered to be interested to act as a relay. The relay devices do not necessarily transmit at the maximum power while relaying data for other devices [6,7,12].

**Figure 1:**
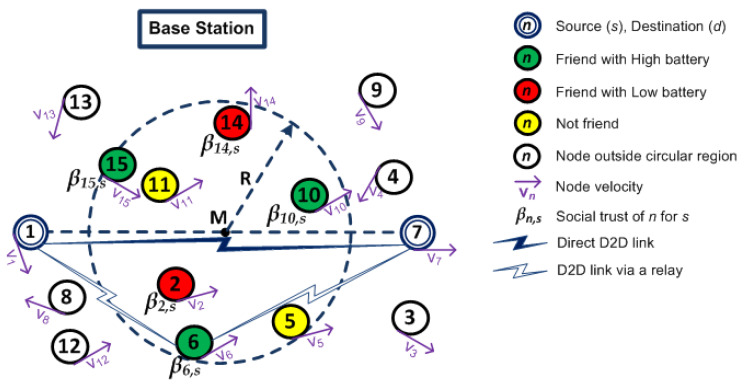
A system model for device-to-device (D2D) communications.

Figure 1 illustrates the search location of a socially trusted relay device that improves the D2D communication between mobile devices. The circles with *n* inside them represent devices in the network, where *n* represents the device number. An arrow in a device represents the direction of mobility and $V_{n}$ underneath represents its velocity. The double-lined circles are source device *s* and destination device *d*. The dashed circle with radius *R* represents a relay selection region which is centered at the midpoint *M* of the distance between *s* and *d*. The circles (with white background) are devices outside relay selection region. The circles (with green background) are friends of source with high battery power levels within the circular region. Similarly, the circles (with red background) are friends of source with low battery power levels. The circles (with yellow background) are devices which are not friends of source. The social trust value that a device *n* has for the source *s* is represented by $\beta_{n,s}$.

We assume that the BS knows location information of devices. The devices in the network have a Global Positioning System (GPS) or a location tracker that periodically updates the BS about their location [3,21]. This helps the BS to locate devices within its coverage area and recommend a list of devices that are located within a circular region for relay selection. A socially trusted relay device is selected among the devices that are located within the circle.

Operator controlled link establishment is used for both direct D2D communication or D2D communication via a relay as envisioned in Reference [26]. When a relay is selected for D2D communications, the same frequency is used for communication between the source to the relay and that from the relay to the destination. The transmission power of D2D communication between mobile devices is determined by the BS. The maximum allowable transmission power, $P_{max}$, is based on network parameters and to avoid interference to neighbouring cellular devices [7,8]. The mobile devices having D2D communications transmit at a power $P_{i,j}\leq$$P_{max}$. A source always transmits at the maximum allowable transmission power i.e. $P_{s,j}$ = $P_{max}$. However, a relay may not transmit at $P_{max}$. We assume the background noise is constant and there is no interference in the system due to high frequency reuse factor [27].

We explain our proposed relay selections schemes in detail, supported by an example scenario, in Section 3.1.

### 2.2. Mobility Model

This section describes the pattern of mobility of users in a network which includes change in location of mobile users over time and the velocity and acceleration at which mobile users change their positions to. In this research, Random Waypoint mobility model [28,29] is used to emulate movement patterns of people in the network. In this mobility model, a node moves from a waypoint in a straight line towards the next waypoint with a certain velocity having minimum velocity of $v_{min}$ and maximum velocity of $v_{max}$. After arriving at the waypoint, the node waits for a constant pause time before selecting next waypoint. The waypoint is selected using Uniform distribution, which resembles the movement of people carrying mobile devices [29,30].

This model is suitable for nodes having limited mobility [31]. For example: a scenario where large number of people come together in a small confined area for specific time duration. This type of scenario are generally present in educational institutions, organizations, industries, or special events like concerts, conferences or exhibitions.

### 2.3. Social Trust Model

This section initially explains why relay devices do not always transmit at its maximum power. Then, it presents social trust model that uses Pareto distribution. This trust model is used to determine transmission power of a relay device.

Generally, mobile devices in communication networks are power constrained. These devices tend to conserve their battery power whenever possible. Even in a cooperative D2D communication network, a relay device may not always transmit at the maximum power possible. This is because when the device transmits at a high power, it drains more of its battery power. A cooperative relay device, which is capable of transmitting at a variable power, prefers to transmit at a low power that is equal to or greater than a minimum threshold value of transmission power. This transmission power is lower than the maximum allowable transmission power.

The transmission power of a relay is assumed to be linearly proportional to the social trust strength between the relay and the source [12]. The transmission power of a relay can be expressed as

(1) $$P_{r}=\left(P_{max}-P_{min}\right)\times \beta_{r,s}+P_{min},$$

where $P_{max}$ is maximum allowable transmission power, $P_{min}$ is minimum allowable transmission power and $\beta_{r,s}$ is social trust value that relay has for source.

The rationale behind this assumption is that when people carrying mobile devices have strong social trust with each other, their willingness for mutual help is comparatively more than those with weak social trust [6,7]. This behaviour of people can be utilized in designing a relay selection scheme of D2D communications. The willingness of helping a device with high social trust is reflected by transmitting at a higher power. The different ways to collect social information of users to estimate social trust values are described in our previous paper [10]. In this paper, we use Pareto distribution to model social trust among users in the network.

Generally, social trust among majority of people in a community are weak. Only few people have high social trust between them [12]. The users having strong social relationship with each other usually have high social trust between them. A mathematical model of a social trust would therefore have a heavy tailed distribution. The authors of References [6,12] have modeled social trust using Pareto distribution to represent a heavy-tailed distribution. Pareto distribution has a probability density function defined as

(2) $$f_{X}\left(x\right)=\frac{\alpha L^{\alpha }x^{-\alpha -1}}{1-{\left(\frac{L}{H}\right)}^{\alpha }},L\leq x\leq H,\mathrm{and}\;\alpha >0,$$

where α, *L* and *H* denote the shape, scale and upper limit parameters, respectively [32]. The value of α determines how fast the distribution tail decays and *L* represents the lower limit of the support. Social trust among devices in the network can be varied by varying α and *L* values, while *H* is kept constant. The random variable X in Equation (2) is the same as the $\beta_{r,s}$ used to represent social trust in Equation (1). Each node knows social trust value it has for other nodes.

In a survey [26], at least 75% of the time, social trust among users in the network are found to be bidirectional. We assume that social trust among users are bidirectional in this paper. When a source can estimate social trust values of other devices, this information helps to determine which devices have the potential to transmit at a high power.

### 2.4. Battery Level Model

This section describes the way battery power level of a device affects its transmission power. We assume that the mobile devices can get information of battery level of other devices in the network. In case of a cellular network, battery level of devices can also be incorporated along with other signalling information, from devices to the BS. The BS then sends the battery information to the source along with other information required for D2D communications. Therefore, the source device is assumed to know the battery power level of devices within its communication range.

We consider that relay mobile devices in the network do not always have full battery power. The relay devices are modelled to have battery power levels ranging from 10% to 100% at an interval of 10. All relay devices are equally likely to have any of the battery levels.

We assume that the transmission power of a relay device also depends upon its battery level. The relay device transmits at a power proportional to the social trust (according to Equation (1)) only when its battery level is greater than a battery threshold ($B_{thres}$). Otherwise, the relay device transmits at a low power possible ($P_{min}$).

Next, we present the communication link model used in this paper for the throughput calculation.

### 2.5. Communication Link Model

#### 2.5.1. Signal-to-Noise-Ratio of a Link

Using SNR values in the probe replies, the source compares between the data rate offered by direct link and D2D link, for the selection of a communication path. The SNR of a direct link can be expressed as

(3) $$\gamma_{s,d}=\frac{P_{s,d}D_{s,d}^{-\theta }}{N},$$

where, $P_{s,d}$ is the transmission power of a signal from the source to the destination, $D_{s,d}$ is the distance between the source and the destination, *N* is the noise power and θ is the pathloss exponent.

#### 2.5.2. Throughput of D2D Communications

According to Shannon’s capacity formula, the maximum reliable data rate of a direct link can be expressed as

(4) $$C_{s,d}=B\log_{2}\left(1+\gamma_{s,d}\right),$$

where, *B* is the bandwidth of the channel.

The data rate of a D2D link having full duplex decode-and-forward (DF) relaying is given by

(5) $$C_{s,r,d}=B\min\left\{\log_{2}\left(1+\gamma_{s,r}\right),\log_{2}\left(1+\gamma_{s,d}+\gamma_{r,d}\right)\right\},$$

where, $\gamma_{s,r}$ is SNR of the signal from the source to the relay, $\gamma_{s,d}$ is SNR of the signal from the source to the destination and $\gamma_{r,d}$ is SNR of the signal from the relay to the destination [6,7,33].

Considering the time spent for probing of candidate relay devices, the throughput of a D2D communication that uses TDMA mechanism can be expressed as

(6) $$T_{s,d}=\frac{C_{s,r,d}\left\{t-\left(\tau \times p\right)\right\}}{t},$$

where, *t* is the time slot duration, τ is the probe duration and *p* is the number of probes [7,8,12]. The throughput decreases with the increase in duration and number of probes. However, to select a relay transmitting with high power, multiple devices are required to be probed.

The source compares the throughput offered by the direct link and that offered by the D2D link to improve performance of D2D communications by selecting a relay device whenever possible.

We consider there are no major obstacles in the propagation path from the source to the destination. We assume the background noise is constant and is interference free. In reality, there might be obstacles in the signal path. The SNR of signal through different candidate relay can be estimated by probing of devices using the protocol presented in the next section.

## 3. Proposed Schemes: Midpoint Relay Selection Using Social Trust and Battery Level

We present the design of relay selection schemes for two-hop D2D communications utilizing social information of mobile users and battery level of mobile devices in the network. A relay is selected among the devices that are located around the midpoint of the distance between the source and the destination. The key difference in the design of relay selection schemes proposed in this paper compared to our previous papers in References [9,10,11] is that our new design also takes into account the battery power of devices while selecting a relay for D2D communications. We propose two variations of midpoint relay selection using social information and battery level of devices. They are:

1. Midpoint Relay Selection Using Social Trust and Battery Level (MRS-ST-BL)
2. Midpoint Relay Selection Using Social Distance and Battery Level (MRS-SD-BL)

This section is mainly focused on the set of tasks performed by different network entities to select a relay for D2D communications, not on details of conventional cellular communications. We present the information exchange between different entities of the network for relay selection, highlighting the use of battery level information of devices during relay selection.

Algorithm 1 illustrates the tasks performed by a BS to select a relay. When a source wants to have a relay for D2D communication, it sends a D2D communication request to the BS which contains the source and the destination identity. In response, BS identifies location of the source and the destination, and calculates the corresponding midpoint *m* using the location information. The BS then identifies the idle nodes that are located within a circular region of radius *R* with the center at the midpoint, and battery level of the idle devices. The BS also determines the maximum transmission power Pmax that is allowable for D2D communication of based on the system parameters (geo-location) and protection requirement of neighbouring cellular devices [7,8]. After that, BS sends maximum allowable transmission power and the list of idle devices and their corresponding battery level to the source. The additional information on battery power level of devices is used compared to MRS probing protocol [10], to increase throughput of D2D communications when devices in the network have varying battery power levels.

The social trust values that the source has for other devices provide an idea of which devices have the potential to transmit at a high power as explained in Section 2.3. Therefore, among the list of idle devices received from the BS, the devices having social trust with the source are identified by the source (e.g., comparing with its contact list). Only the devices that have social trust values above certain social threshold are selected from the list. The way of filtering of devices for relay selection are different for MRS-ST-BL and MRS-SD-BL, and are implemented as in Algorithm 2 for MRS-ST-BL and Algorithm 3 for MRS-SD-BL.

In the proposed MRS-ST-BL, a source initially filters the socially trusted devices above social threshold. The source sequentially probes at most *l* devices from the list having large social trust values. The probed device transmits at a power proportional to the social trust only when the battery level of the device is greater than or equal to battery threshold ($B_{\mathrm{thres}}$). Otherwise, the device transmits at a lowest power possible.

In the proposed MRS-SD-BL, a source initially filters at most *l* devices nearest to the midpoint and probes the devices having social trust above social threshold. The transmission power of a probed device also depends upon the battery level of the device and social trust as in MRS-ST-BL.

As a reply to a probe, each of the candidate relay devices, individually responds to the source with SNR of the probe signal they have received and also forwards the probe message to the destination as implemented in Algorithm 4. Each of the relaying devices transmits at a power proportional to the social trust when the battery level on the device ≥$B_{\mathrm{thres}}$. Otherwise, the device transmits at the low power possible.

The source receives the probe reply from the candidate relay devices and destination. The destination sends SNR for each of the received probe packet and sends to the source as in Algorithm 6 (according to Equation (3)). When the data rate offered by the direct link is less than that by a D2D link, a relay is selected. Upon receiving user data from the source, the relay forwards the user data to the destination as illustrated in Algorithm 5. The destination also sends user data to the source via the selected relay.

### 3.1. An Example Scenario of Our Proposed Schemes

Let us consider that the mobile devices are under the coverage of a base station (BS) as shown in Figure 1. Each mobile devices are moving with different velocities at random direction. We assume that the BS knows location and battery level information of devices in the network. Suppose a mobile device (source) wants to have D2D communication with another mobile device (destination). The source can either have direct D2D communication with the destination or select a relay for D2D communication. The source selects a direct D2D communication only when its data rate is higher compared to that offered by a D2D communication via a relay. We propose MRS-ST-BL and MRS-SD-BL relay selection schemes that maximizes throughput of D2D communications by taking location, battery level and social trust information into account for relay selection.    
**Algorithm 1:** Tasks performed at Base Station// Given a source and a destination pair, BS determines candidate relay nodes and their corresponding battery power level and maximum allowable transmission power.Input: *s*, *d*, *R* and battery percentage of nodesOutput: $n_{\mathrm{circle}}$ along with battery percentage and location, and $P_{\max}$1:Receive D2D request from *s*2:Calculate Midpoint, *m* = Midpoint(*s*, *d*)3:**for**radius = 1: *R* **do**4:    Identify $n_{\mathrm{circle}}$ = NodesWithinCircle(*m*, radius)5:    Identify battery percentage of devices in $n_{\mathrm{circle}}$6:**end for**7:Determine $P_{\max}$8:Send $n_{\mathrm{circle}}$ along with battery percentage and location, and $P_{\max}$ to *s*
**Algorithm 2:** Tasks performed at Source for MRS-ST-BL// This algorithm consists of sequence of tasks performed by a node for midpoint relay selection using social trust and battery power. If a relay is to be used, it selects a relay that maximizes the throughput of D2D communication.Input: *s*, *d*, $n_{\mathrm{circle}}$, battery level of nodes listed in $n_{\mathrm{circle}}$, location of nodes listed in $n_{\mathrm{circle}}$, $S_{\mathrm{thres}}$, $B_{\mathrm{thres}}$, $P_{\max}$, *l*, *T* and τOutput: $t_{s},r,d$
1:**if** len($n_{\mathrm{circle}}$) = 0 **then**2:    Calculate $C_{s,d}$ as in equation 43:    $t_{s},r,d$ = $C_{s,d}$4:**else**5:    **if** Friends in $n_{\mathrm{circle}}$ = 0 **then**6:        Relay = Nearest to midpoint7:        $t_{s},r,d$ = $C_{s,r,d}$8:    **else**9:        Identify ${n_{S}}_{\mathrm{thres}}$10:        **if** len(${n_{S}}_{\mathrm{thres}}$) = 0 **then**11:           Relay = Nearest to midpoint12:           $t_{s,r,d}$ = $C_{s,r,d}$13:        **else**14:            $n_{\mathrm{battery}}$ = Identify nodes having battery level above $B_{\mathrm{thres}}$15:           **if** len($n_{\mathrm{battery}}$) >1 **then**16:               $n_{\mathrm{battery}}$ = *l* nodes with high social trust values17:           **end if**18:           Sequentially probe nodes in $n_{\mathrm{battery}}$ and *d*19:           **for** *i* = 1: len($n_{\mathrm{battery}}$ ) **do**20:               Power of ${n_{S}}_{\mathrm{thres}}$(*i*) = Transmission power proportional to social trust21:               Calculate $C_{s,r,d}$ through each of nodes in ${n_{S}}_{\mathrm{thres}}$ as in equation 522:           **end for**23:           Select maximum $C_{s,r,d}$ = $max(C_{s,r,d})$24:           **if** maximum $C_{s,r,d}$>$C_{s,d}$ **then**25:               D2D via a relay is selected26:               $t_{s,r,d}=max\left(C_{s,r,d}\right)\times (T-(\tau \times len(n_{\mathrm{battery}}$)))27:           **else**28:               Direct D2D is selected29:               $t_{s,r,d}=C_{s,d}\times (T-(\tau \times len(n_{\mathrm{battery}}$)))30:           **end if**31:        **end if**32:    **end if**33:**end if**


When a source (let us say device 1) wants to have D2D communication with a destination (let us say device 7), it sends a D2D request to the BS. The BS sends list of devices (let us say 2, 5, 6, 10, 11, 14, 15) within a circular region of radius *R* centered at the midpoint between source and destination, battery of the devices along with other necessary information for D2D communications.

In our proposed scheme MRS-ST-BL, the source initially filters the devices that have social trust above social threshold (let us say $S_{\mathrm{thres}}$ = 0.3) and also have battery above battery threshold (let us say $B_{\mathrm{thres}}$ = 30%). Let us consider devices 5 and 11 are not friends of source. These devices are not considered as potential relays. Therefore, they are filtered out. Among the list of remaining devices, let us consider devices 2 and 14 have battery level less than $B_{\mathrm{thres}}$. These devices (2 and 14) are also not considered for relay selection because they may not relay the data successfully. The source then filters at most *l* (let us say probe limit = 10) devices that have large social trust values among the remaining devices in the list. In the considered scenario, devices 6, 10 and 15 are sequentially probed by the source. The source compares the data rate offered by each of them. Let us say, device 6 offers the maximum data rate. This data rate is compared against the data rate of direct communication. If the data rate through the device 6 is higher than that of direct communication, then device 6 is selected as a relay. Otherwise, the source communicates directly with the destination.    
**Algorithm 3:** Tasks performed at Source for MRS-SD-BL// This algorithm consists of sequence of tasks performed by a node for midpoint relay selection using social distance and battery power. If a relay is to be used, it selects a relay that maximizes the throughput of D2D communication.Input: *s*, *d*, $n_{\mathrm{circle}}$, battery level of nodes listed in $n_{\mathrm{circle}}$, location of nodes listed in $n_{\mathrm{circle}}$, $S_{\mathrm{thres}}$, $B_{\mathrm{thres}}$, $P_{\max}$, *l*, *T* and τOutput: $t_{s},r,d$1:**if** len($n_{\mathrm{circle}}$) = 0 **then**2:    Calculate $C_{s,d}$ as in equation 43:    $t_{s},r,d$ = $C_{s,d}$4:**else**5:    **if** Friends in $n_{\mathrm{circle}}$ = 0 **then**6:        Relay = Nearest to midpoint7:        $t_{s},r,d$ = $C_{s,r,d}$8:    **else**9:         $n_{\mathrm{battery}}$ = Identify nodes having battery level above $B_{\mathrm{thres}}$10:        **if** len($n_{\mathrm{battery}}$) = 0 **then**11:           Relay = Nearest to midpoint12:           $t_{s},r,d$ = $C_{s,r,d}$13:        **else**14:           **if** len($n_{\mathrm{battery}}$) >1 **then**15:               $n_{\mathrm{battery}}$ = *l* nodes nearest to midpoint16:           **end if**17:           Identify node above social threshold, ${n_{S}}_{\mathrm{thres}}$18:           **if** len(${n_{S}}_{\mathrm{thres}}$) = 0 **then**19:               Relay = Nearest to midpoint20:               $t_{s},r,d$ = $C_{s,r,d}$21:           **else**22:               Sequentially probe nodes in ${n_{S}}_{\mathrm{thres}}$ and *d*23:               **for** *i* = 1: len(${n_{S}}_{\mathrm{thres}}$) **do**24:                   Power of ${n_{S}}_{\mathrm{thres}}$(*i*) = Transmission power proportional to social trust25:               **end for**26:               Calculate $C_{s,r,d}$ through each of nodes in ${n_{S}}_{\mathrm{thres}}$ as in equation 527:               Select maximum $C_{s,r,d}$ = $max(C_{s,r,d})$28:               **if** $max(C_{s,r,d})$>$C_{s,d}$ **then**29:                   D2D via a relay is selected30:                   $t_{s,r,d}=max\left(C_{s,r,d}\right)\times (T-(\tau \times len({n_{S}}_{\mathrm{thres}}$)))31:               **else**32:                   Direct D2D is selected33:                   $t_{s,r,d}=C_{s,d}\times (T-(\tau \times len({n_{S}}_{\mathrm{thres}}$)))34:               **end if**35:           **end if**36:        **end if**37:    **end if**38:**end if**
**Algorithm 4:** Tasks performed at Candidate Relay Nodes// This algorithm consists of sequence of tasks performed by candidate relay node for relay selection.Input: *s*, *d*, $P_{\max}$ and probeOutput: SNR values1:Receive probe message from *s*2:Send SNR value of probe received to *s*3:Check the battery power level4:**if** battery <$B_{\mathrm{thres}}$ **then**5:    Transmission power = Minimum transmission power possible6:**else**7:    Transmission power = Transmission power proportional to social trust8:**end if**9:Send probe to *d* with transmission power as calculated above

**Algorithm 5:** Tasks performed at a Relay Node
// This algorithm consists of sequence of tasks performed by a relay node in D2D communications.
Input: *s*, *d*, $P_{\max}$ and user data
Output: relay data
1:Receive user data from *s*2:Check the battery power level3:**if** battery <$B_{\mathrm{thres}}$ **then**4:    Transmission power = Minimum transmission power possible5:
**else**
6:    Transmission power = Transmission power proportional to social trust7:
**end if**
8:Send probe to *d* with transmission power as calculated above


**Algorithm 6:** Tasks performed at Destination
// This algorithm consists of sequence of tasks performed by destination node for relay selection.
Input: probe and data
Output: SNR values, data
1:Receive probe messages from *s* and *r*2:Send SNR values of probe received from *s* and *r* to *s*3:Send probe reply to *s*4:Receive data from *r* sent by *s*5:Send user data to *s* via *r*


In our second proposed scheme MRS-SD-BL, the source initially filters the devices that are friends of source and have battery above battery threshold ($B_{\mathrm{thres}}$). Let us say the selected devices are 6, 10 and 15. If the number of selected devices are more than a probe limit, it filters at most *l* (probe limit) devices that are nearest to midpoint of the distance between source and destination. Then, the source filters devices that have social trust above social threshold ($S_{\mathrm{thres}}$) to probe sequentially. Let us say, only devices 15 and 6 have social trust above social threshold. After probing them and the destination directly, the source compares data rate offered by each of them. If data rate of device 6 is higher than that of device 15 and direct communication, device 6 is selected as a relay.

Next, we illustrate the the communication protocol for the proposed relay selection schemes explaining the information exchange between network entities during relay selection.

## 4. Proposed Protocol: Probing of Devices

**Figure 2:**
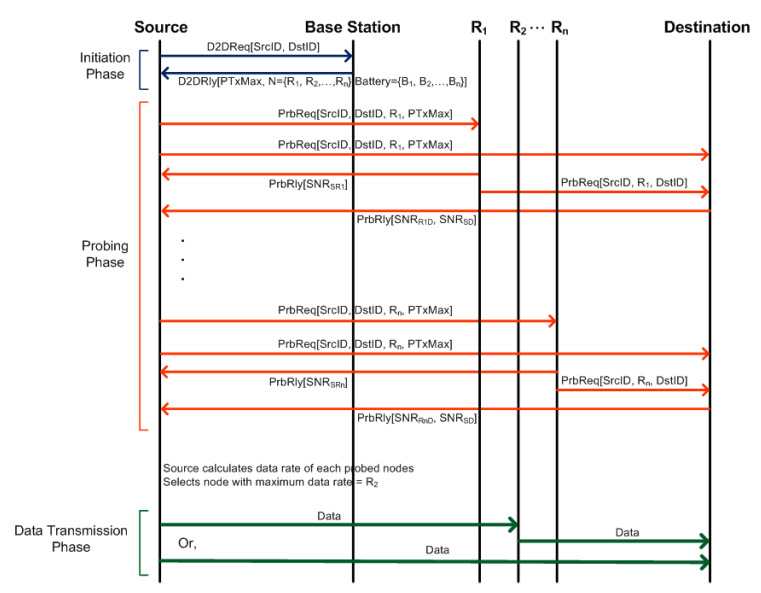
Probing for relay selection.

Probes are short messages sent across devices in the network to exchange information between them. To select a relay, probing messages are exchanged between a source, a base station, other devices in proximity, and a destination. The design of our communication protocol for relay selection in D2D communications is shown in Figure 2. It consists of three phases: Initiation phase, Probing phase and Data transmission phase.

The initiation phase begins when a source wants to have a relay for D2D communication. The source sends a probe (D2D request) to the BS consisting of source and destination identity. As a response, the BS sends a message to the source containing a list of idle devices that are located within certain search radius (*R*) along with corresponding battery level and maximum allowable transmission power ($P_{\max}$) that can be used for D2D communications as implemented in Algorithm 1. The tasks performed by BS in this proposed scheme is similar to that of MRS-ST in Reference [10]. However, the difference in this paper is that the BS sends additional battery level information of devices to the source.

Upon receiving message from the BS, the source determines the socially trusted devices in the list using its contact list and initiates probing of those devices sequentially [7].

In the probing phase, a source probes the nearby socially trusted devices to learn about link conditions for relaying data from the source to the destination as implemented in Algorithm 2 for MRS-ST-BL and Algorithm 3 for MRS-SD-BL. The probe message contains source identity, destination identity, probed device identity and maximum allowable transmission power. The message is also received by the destination. As a reply to a probe, each of the devices which are available for relaying data, individually responds to the source with SNR of the probe signal they have received and also forwards the probe message to the destination. The destination calculates SNR for each of the received probing packets and sends the SNR values to the source. The source calculates estimated data rate offered by the probed devices based upon link conditions and transmission power, and selects a device which offers maximum rate.

The source takes into account time wasted while probing devices. The more candidate devices to be probed, the more time is wasted for real communications. The source estimates the throughput offered by that selected device and compares it with the direct communication. Data transmission phase starts after the source decides whether to use the selected device as a relay or not. If a relay is used for D2D communications, the source sends the data to the relay, which is then forwarded to the destination.

### Theoretical Probing Protocol Analysis

This section presents the probing overhead comparison of HRS, MRS-ST-BL and MRS-SD-BL. The probing overheads of each of the schemes are compared by calculating the average number of messages exchanged before user data transmission for each of them. The average probe number (*p*) can be used to quantify the average time taken for probing. The less the time spent for probing in a scheme, the higher the throughput can be achieved by the scheme.

Figure 2 shows the exchange of messages between a source, candidate relay nodes and a destination in MRS-ST-BL and MRS-SD-BL. Similar messages are also exchanged in HRS. In all these schemes, during the D2D initiation phase, a source sends a D2D request message to a BS. The BS sends a D2D reply back to the source. During the probing phase, each candidate relay nodes are probed to learn about physical link conditions. Probing of a candidate relay node requires exchange of 4 messages: from source to relay, relay to source (for SNR value from *s* to *r*), relay to destination, destination to source (for SNR value from *s* to *d*, and SNR value from *r* to *d*). HRS also requires similar message exchange while probing. Therefore, the average number of messages exchanged in MRS-ST-BL, MRS-SD-BL and HRS before D2D communication establishment are

(7) $$\begin{array}{cc} M & =2+4\times p, \end{array}$$

which shows that the number of messages exchanged is proportional to the number of devices being probed. The graphical illustration of average number of probes for these schemes are in Section 5.3.2.

Now we calculate the number of nodes probed, by first defining the node density and number of nodes in a circular region. Using width of a network *W* and $n_{\mathrm{total}}$ number of devices in the network, node density $n_{\mathrm{density}}$ can be calculated as

(8) $$\begin{array}{c} n_{\mathrm{density}}=n_{\mathrm{total}}/W^{2}. \end{array}$$

Within a circular region of radius *R*, the average number of devices that can be a relay are

(9) $$\begin{array}{cc} n_{\mathrm{circle}} & =R\times n_{\mathrm{total}}/W^{2} \end{array}$$

Let *x* be the fraction of devices which are friends of source and *y* be the fraction of friend devices whose social trust values are above the social threshold. Its value can be calculated using the social distribution discussed in Section 2.

In HRS, the number of devices having social trust above social threshold are

(10) $$\begin{array}{cc} n_{\mathrm{HRS}} & =x\times y\times \frac{R\times n_{\mathrm{total}}}{W^{2}} \end{array}$$

which is the same as the average number of devices to be probed, $p_{\mathrm{HRS}}$.

In MRS-ST-BL, the number of devices above the social threshold are

(11) $$\begin{array}{c} n_{\mathrm{MRS}-\mathrm{ST}-\mathrm{BL}}=x\times y\times n_{\mathrm{circle}} \end{array}$$

Among the devices above social threshold, it will filter the devices having battery level above battery threshold ($B_{thres}$). Let *z* be the fraction of devices having battery level above $B_{thres}$. Then the number of devices within circular region having battery level above $B_{thres}$ are

(12) $$\begin{array}{c} n_{\mathrm{MRS}-\mathrm{ST}-\mathrm{BL}}=x\times y\times z\times n_{\mathrm{circle}} \end{array}$$

At most *l* devices are probed in MRS-ST-BL. $n_{\mathrm{MRS}-\mathrm{ST}-\mathrm{BL}}$ devices are arranged with their social trust in descending order. Then, the average number of devices to be probed are

(13) $$\begin{array}{c} p_{\mathrm{MRS}-\mathrm{ST}-\mathrm{BL}}=\left\{\begin{array}{cc} l & \mathrm{if}\;l\;<\;n_{\mathrm{MRS}-\mathrm{ST}-\mathrm{BL}};\\ n_{\mathrm{MRS}-\mathrm{ST}-\mathrm{BL}} & \mathrm{otherwise}. \end{array}\right. \end{array}$$

In MRS-SD-BL, friends having battery level above battery threshold ($B_{thres}$) are initially filtered as

(14) $$\begin{array}{c} n_{\mathrm{MRS}-\mathrm{SD}-\mathrm{BL}}=x\times z\times n_{\mathrm{circle}} \end{array}$$

At most *l* devices are probed in MRS-SD-BL. Therefore, the filtered devices are arranged according to distance from the midpoint in ascending order. At most *l* devices nearest to the midpoint are filtered again as

(15) $$\begin{array}{c} n_{\mathrm{MRS}-\mathrm{ST}-\mathrm{BL}-\mathrm{probelimit}}=\left\{\begin{array}{cc} l & \mathrm{if}\;l\;<\;n_{\mathrm{MRS}-\mathrm{SD}-\mathrm{BL}};\\ n_{\mathrm{MRS}-\mathrm{ST}-\mathrm{BL}} & \mathrm{otherwise}. \end{array}\right. \end{array}$$

Then, the average number of devices above the social threshold is equal to average number of devices to probed in MRS-SD-BL. It is expressed as

(16) $$\begin{array}{c} p_{\mathrm{MRS}-\mathrm{SD}-\mathrm{BL}}=n_{\mathrm{MRS}-\mathrm{ST}-\mathrm{BL}-\mathrm{probelimit}}\times y \end{array}$$

The average number of probes in MRS-SD-BL is less than that of MRS-ST-BL, when social trust values for the link between source and the candidate relay nodes are low. This is because, when the social trust values of at most *l* devices are low, number of devices having trust values above social threshold obviously becomes less in MRS-SD-BL.

The probing frequency can be reduced for selection of a relay when devices in the network have limited mobility and the link conditions are stable. However, if the devices in the network have high mobility and link conditions are changing, the probing of devices should be done more frequently to select a relay.

## 5. Performance Analysis of Proposed Schemes: MRS-ST-BL and MRS-SD-BL

### 5.1. Simulation Setup

Table 1 shows the details of the simulation setup used in our research. A custom simulation model of HRS, M-Nearest, M-Nearest_MaxTx, MRS-ST and MRS-SD was developed in Octave [29]. One thousand devices are distributed uniformly at random across a square network area, with widths of 100 and 1000 m. Random Waypoint mobility model is used to emulate movement patterns of people as close as possible. The mobility model is implemented using BonnMotion [34].

We are considering a scenario where people carrying mobile devices are walking within a certain area. Therefore, the speed of devices is varied between 0–2 m/s and have maximum pause time of 5 s. The social trust value between two devices follow Pareto distribution. Different social trust scenarios among devices in the network are achieved by varying the Shape and Scale parameters. Source and destination are selected randomly from the 1000 devices. We set the transmission power of a source device $P_{s,d}$ = 20 dBm, noise power *N* = −114 dBm, pathloss exponent θ = 4, bandwidth of channel *B* = 1 MHz as in References [12,35]. We assume the duration of a time slot for D2D communications *T* = 1 s and that for a probe τ = 0.01 s [5]. The mobile devices are considered to have 10 different battery percentage levels with the difference of 10 between them. The battery percentage of a device is selected randomly. Each simulation runs for 1000 s. In this analysis, the maximum allowable transmission power for a relay is considered to be equal to transmission power of source. The results reported for each of the schemes are time average of the throughput.

Section 5.3 presents the results various relay selection schemes analyzed based on this simulation setup.

### 5.2. Schemes Compared

This section presents the short description of all schemes analyzed in this paper. The schemes we have analyzed are as follows:

- Direct Communication (Direct): A scheme in which a source device directly transmits to a destination device. This scheme does not use a relay to assist D2D communication between the source and the destination.
- Hybrid Relay Selection with Full Battery (HRS-FB): A scheme same as HRS scheme proposed by Pan and Wang [12]. The relay device always has a full battery level and transmits at a power proportional to the social trust.
- Hybrid Relay Selection Without Full Battery (HRS-WFB): A modified version of HRS [12]. This scheme is used to analyze the performance of HRS when a relay has varying battery level. In this scheme, a relay device transmits at a power proportional to the social trust only when the battery level on the device is above certain threshold value ($B_{thres}$). Otherwise, the relay device transmits at a lowest power (not proportional to the social trust). However, the battery level of devices are not taken into account in the relay selection design.
- Hybrid Relay Selection with Different Battery Levels (HRS-BL): A modified version of HRS [12]. This scheme is also used to analyze the performance of HRS when a relay has varying battery level. This scheme is similar to HRS-WFB, except the battery level of devices are taken into account in the relay selection design.
- Nearest to Midpoint with Full Battery (M-Nearest-FB): A scheme which selects a relay device nearest to the midpoint. The relay device always has a full battery level and transmits at a power proportional to the social trust.
- Nearest to Midpoint Without Full Battery (M-Nearest-WFB): A scheme which selects a relay device nearest to the midpoint. It is used to analyze the performance of M-Nearest when a relay has varying battery level. In this scheme, a relay device transmits at a power proportional to the social trust only when battery level on the device is above certain threshold value ($B_{thres}$). Otherwise, the relay device transmits at a lowest power (not proportional to the social trust). However, the battery level of devices are not taken into account in the relay selection design.
- Nearest to Midpoint with Different Battery Levels (M-Nearest-BL): A scheme which selects a relay device nearest to the midpoint. It is used to analyze the performance of M-Nearest when a relay has varying battery level. This scheme is similar to M-Nearest-WFB, except the battery level of devices are taken into account in the relay selection design.
- Nearest to Midpoint with Maximum Transmission Power (M-Nearest_MaxTx): A scheme which represents an ideal relay selection (practically not feasible). It is used as a benchmark scheme that provides the upper bound for the throughput of D2D communication. This scheme selects a relay device nearest to the midpoint. The relay device always has a full battery and also transmits at the maximum power.
- Midpoint Relay Selection using Social Trust with Full Battery (MRS-ST-FB): A scheme same as MRS-ST [10]. The relay device always has a full battery level and transmits at a power proportional to the social trust.
- Midpoint Relay Selection using Social Trust Without Full Battery (MRS-ST-WFB): A modified version of MRS-ST [10]. It is used to analyze the performance of MRS-ST when a relay has varying battery level. In this scheme, a relay device transmits at a power proportional to the social trust only when the battery level on the device is above certain threshold value ($B_{thres}$). Otherwise, the relay device transmits at a lowest power (not proportional to the social trust). However, the battery level of devices are not taken into account in the relay selection design.
- Midpoint Relay Selection using Social Trust and Battery Level (MRS-ST-BL): Our proposed scheme which considers battery level of devices together with location and social information in the relay selection design. In this scheme, a relay device transmits at a power proportional to the social trust only when battery level on the device is above certain threshold value ($B_{thres}$). Otherwise, the relay device transmits at a lowest power (not proportional to the social trust).
- Midpoint Relay Selection using Social Distance with Full Battery (MRS-SD-FB): A scheme same as MRS-SD [10]. The relay device always has a full battery and transmits at a power proportional to the social trust.
- Midpoint Relay Selection using Social Distance Without Full Battery (MRS-SD-WFB): A modified version of MRS-SD [10]. It is used to analyze the performance of MRS-SD when a relay has varying battery level. In this scheme, a relay device transmits at a power proportional to the social trust only when battery level on the device is above certain threshold value ($B_{thres}$). Otherwise, the relay device transmits at a lowest power (not proportional to the social trust). However, the battery level of devices are not taken into account in the relay selection design.
- Midpoint Relay Selection using Social Distance and Battery Level (MRS-SD-BL): Our proposed scheme which considers battery power level of devices together with location and social information in the relay selection design. In this scheme, a relay device transmits at a power proportional to the social trust only when battery level on the device is above certain threshold value ($B_{thres}$). Otherwise, the relay device transmits at a lowest power (not proportional to the social trust). This scheme is different from MRS-ST-BL in the way it filters devices for selection of a relay.

### 5.3. Results

#### 5.3.1. Impact of Different Battery Levels of Relays on Throughput

This section presents the impact analysis of different battery power levels of relays on the throughput of various relay selection schemes in networks. The analysis is done for different social trust scenarios and different node densities. The key observation from our analysis is that the throughput of existing relay selection schemes decrease when the relays have different battery levels. In such scenarios, our proposed schemes MRS-ST-BL and MRS-SD-BL significantly enhance the throughput of D2D communications.

**Figure 3:**
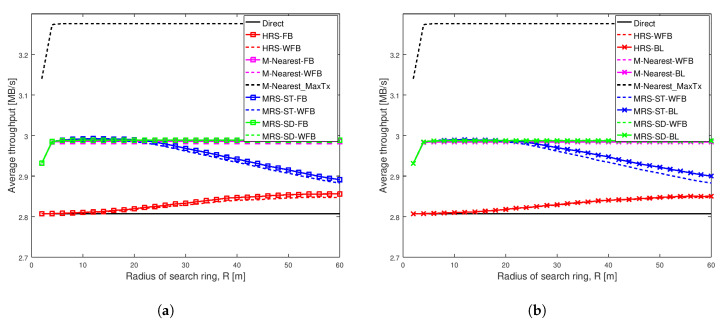
Average throughput of different schemes when Shape = 1.001 and Scale = 0.001 in a network having width of 100 m. (**a**) Different battery power levels not considered in relay selection; (**b**) Different battery power levels considered in relay selection.

**Figure 4:**
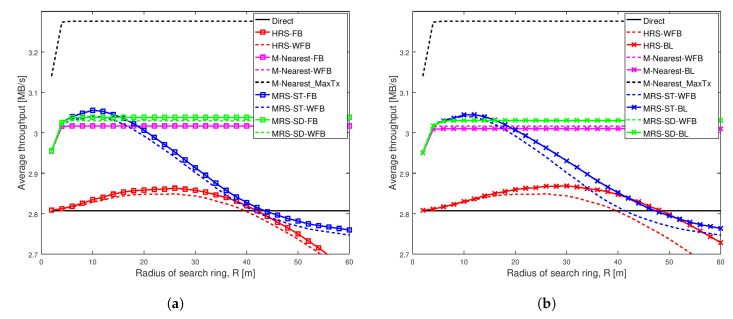
Average throughput of different schemes when Shape = 1.01 and Scale = 0.01 in a network having width of 100 m. (**a**) Different battery power levels not considered in relay selection; (**b**) Different battery power levels considered in relay selection.

**Figure 5:**
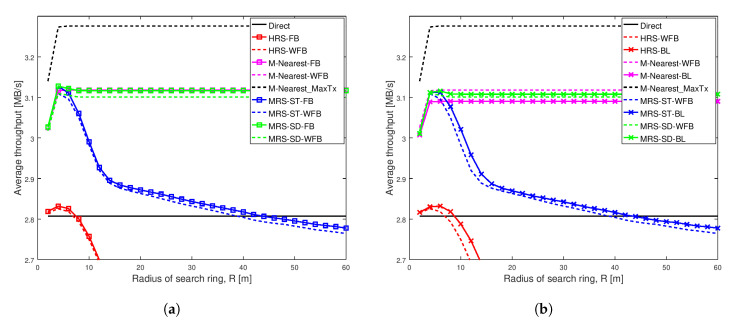
Average throughput of different schemes when Shape = 1.1 and Scale = 0.1 in a network having width of 100 m. (**a**) Different battery power levels not considered in relay selection; (**b**) Different battery power levels considered in relay selection.

**Figure 6:**
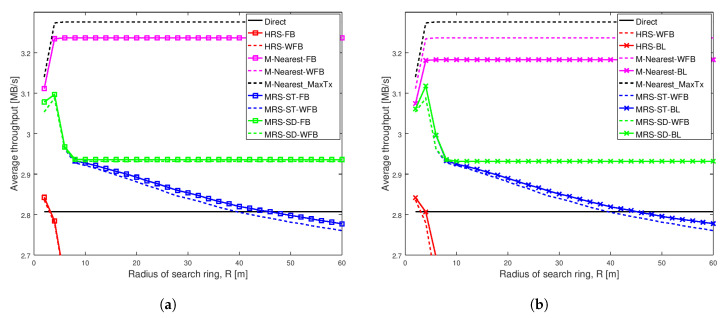
Average throughput of different schemes when Shape = 2 and Scale = 0.5 in a network having width of 100 m. (**a**) Different battery power levels not considered in relay selection; (**b**) Different battery power levels considered in relay selection.

**Figure A1:**
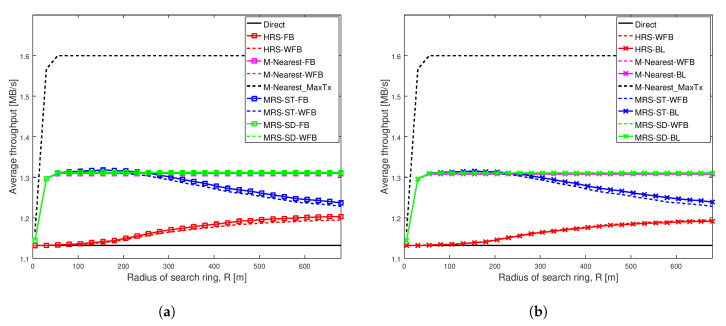
Average throughput of different schemes when Shape = 1.001 and Scale = 0.001 in a network having width of 1000 m. (**a**) Different battery power levels not considered in relay selection; (**b**) Different battery power levels considered in relay selection.

**Figure A2:**
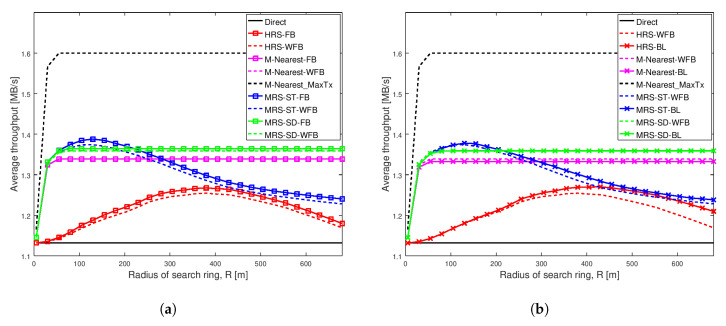
Average throughput of different schemes when Shape = 1.01 and Scale = 0.01 in a network having width of 1000 m. (**a**) Different battery power levels not considered in relay election; (**b**) Different battery power levels considered in relay selection.

**Figure A3:**
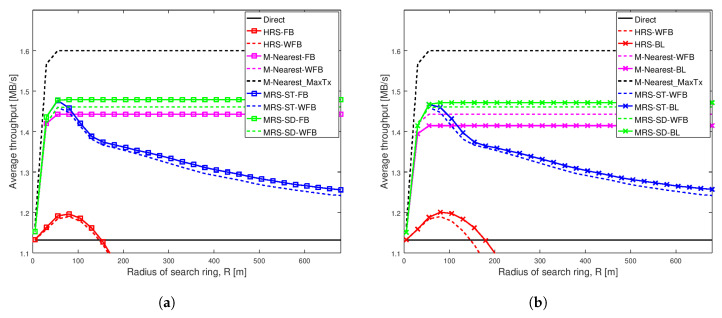
Average throughput of different schemes when Shape = 1.1 and Scale = 0.1 in a network having width of 1000 m. (**a**) Different battery power levels not considered in relay selection; (**b**) Different battery power levels considered in relay selection.

**Figure A4:**
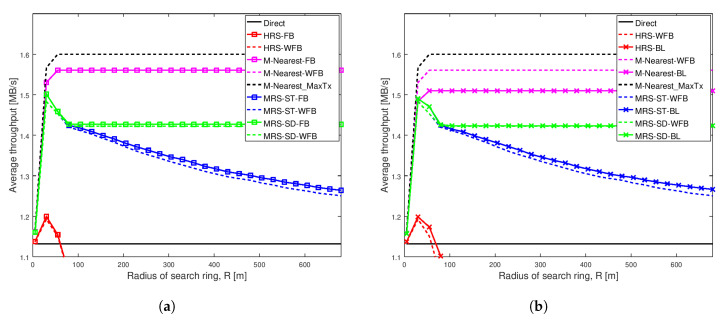
Average throughput of different schemes when Shape = 2 and Scale = 0.5 in a network having width of 1000 m. (**a**) Different battery power levels not considered in relay selection; (**b**) Different battery power levels considered in relay selection.

Figure 3, Figure 4, Figure 5 and Figure 6 show the throughput comparison at different social trust scenarios for a high node density network (having width of 100 m). In all these figures, average throughput is on the y-axis and radius of search ring is on the x-axis. The search radius ranges from 2 m up to 60 m and simulation data are recorded at an interval of 2 m. Each scheme analyzed is represented by a line. We also conducted the analysis for different social trust scenarios in a network having width of 1000 m (low node density network). The results are presented in Appendix A.1. Figure A1, Figure A2, Figure A3 and Figure A4 show the average throughput variation for all the schemes are similar to that for a network having width of 100 m. The remaining of this section presents the analysis for network having width of 100 m.

For each of the relay selection schemes, there are three variations analyzed. A line with squares represents throughput of a scheme that has a relay with full battery power. A dashed line represents throughput of the scheme that has a relay with varying battery level. A line with crosses represents throughput of the scheme that has a relay with varying battery level, but we adjust the scheme to take varying battery level of the relay into account.

The left graph in each of these figures show the average throughput comparison of each scheme with and without full battery. We analysed the average throughput of MRS-ST and MRS-SD (our previous paper [10]), HRS [12] and M-Nearest schemes using relays with full battery against the schemes using relays with different battery levels. The right graph in each of these figures compares the average throughput of our proposed schemes MRS-ST-BL and MRS-SD-BL, when the relay has different battery power levels. The performance of the proposed schemes are compared against the throughput of MRS-ST, MRS-SD, HRS and other generic schemes without battery awareness in relay selection, but relays have different battery levels.

Figure 3 depicts the average throughput of various schemes as the search radius increases for a very weak social trust scenario (Shape = 1.001, Scale = 0.001) among users in a network with high node density. Each line in the graph represents the average throughput of a scheme. The key result from this figure is that the different battery levels of relays do not impact the performance of any of the relay selection schemes analyzed, when social trust among the users in the network are very weak. Additionally, the proposed schemes MRS-ST-BL and MRS-SD-BL, which take battery level of devices into consideration while selecting a relay, do not show significant improvement in throughput of D2D communications, when social trust among users in the network are very weak.

Figure 3a illustrates the average throughput comparison of existing schemes MRS-ST and MRS-SD (our previous paper [10]), HRS [12] and generic schemes, with the increase in radius of search ring. The average throughput of these schemes with relays having full battery are compared against throughput of the schemes with relays having different battery levels. The graph shows the average throughput of all schemes initially increases with the increase in search radius, except for Direct scheme which remains constant. The throughput of MRS-ST with a relay having full battery power (the blue line with square) and a relay having different battery power (the dashed blue line) gradually decreases for large search radius as expected. The reason is that, with the increase in search radius, more devices are being probed, and also the length of link from a source to a relay and that from the relay to a destination increases. For large search radius, the throughput of MRS-ST and HRS with varying battery level are reduced compared to the throughput of MRS-ST and HRS with full battery. For other schemes, the throughput remains unchanged for large values of search radius. Next, we present the performance of our proposed schemes MRS-ST-BL and MRS-SD-BL to overcome such reduction in throughput.

Figure 3b illustrates the average throughput of various relay selection schemes as the search radius increases. A relay for each of the schemes have different battery power levels. The proposed schemes MRS-ST-BL and MRS-SD-BL, which take battery power of devices into consideration for selection of a relay are compared against existing schemes MRS-ST, MRS-SD, HRS and M-Nearest schemes with relays having different battery power levels. We also analyzed the performance of modified version of HRS, HRS-RP, in which relay selection is done considering battery power of devices. The important result is that the throughput of our proposed scheme MRS-ST-BL is greater than that of MRS-ST-WFB for large values of search radius.

Figure 4 depicts the average throughput of different schemes as the search radius increases for a weak social trust scenario (Shape = 1.01, Scale = 0.01) among users in a network with high node density. The key observation from this figure is that the varying battery power level of a relay significantly affect the performance of all relay selection schemes, when social trust among the users in the network are weak. Additionally, our proposed scheme MRS-ST-BL significantly increases the throughput of D2D communications when relay devices have varying battery levels.

Figure 4a illustrates the average throughput comparison of existing schemes MRS-ST and MRS-SD (our previous work [10]), HRS [12] and generic schemes, with the increase in radius of search ring. The average throughput of these schemes with a relay having full battery power is compared against throughput of the schemes with a relay having varying battery power.

Figure 4a shows that the average throughput of all schemes initially increases with the increase in search radius, except for Direct scheme which remains constant. The blue line with squares represents the throughput of MRS-ST with a relay having full battery and the dashed blue line represents the throughput of MRS-ST with a relay having varying battery level. The green line with squares represents the throughput of MRS-SD with a relay having full battery and the dashed green line represents a relay with varying battery level. The red line with squares represents the throughput of HRS with a relay having full battery and the dashed red line represents a relay having varying battery level. The throughput of MRS-ST and HRS, that have relay with and without varying battery power levels, gradually decrease for large search radius. This is because with the increase in search radius, more devices are being probed and also the length of link from a source to a relay and that from the relay to a destination increases. Interestingly, the throughput of MRS-ST, MRS-SD and HRS significantly decreases when a relay has varying battery power compared to the schemes when relays have full battery. The throughput of M-Nearest is not affected by battery level of a relay.

Figure 4b illustrates the average throughput comparison of different relay selection schemes with a relay having different battery levels. Our proposed schemes MRS-ST-BL and MRS-SD-BL, which take battery level of devices into consideration for selection of a relay are compared against existing schemes MRS-ST, MRS-SD, HRS and generic schemes with a relay having different battery power levels. We also analyzed the performance of modified version of HRS, HRS-RP, in which relay selection is done considering battery level of devices.

The main result from Figure 4b is that the throughput of our proposed scheme MRS-ST-BL is highest among all others. For small radius, the throughput of MRS-ST-BL is same as that for MRS-ST-WFB when search radius is small. However, with the increase in search radius, the throughput of the proposed scheme MRS-ST-BL is significantly higher than that of MRS-ST-WFB. When the search radius is very large, the difference in throughput is reduced. This is because when search radius is large, the link length from source to relay and that from the relay to destination affects more on SNR values than transmission power of relay. All other schemes attains lower throughput than that of MRS-ST-WFB. The throughput of our proposed scheme MRS-SD-BL is not improved compared to that of MRS-SD-WFB. This is because of the way MRS-SD-BL filters devices to select a relay.

Figure 5 shows the same scenario as Figure 4, except the social trust scenario is stronger with Shape = 1.1 and Scale = 0.1. The results are almost the same as in Figure 4.

Figure 6 depicts average throughput comparison of different schemes for a strong social trust scenario (Shape = 2, Scale = 0.5) among users in a network with high node density. The key result from this figure is that network with strong social trust between users have no significant difference in the performance of all relay selection schemes, when the relays have different battery power levels. Additionally, the figure shows that when social trust between the users are strong, our proposed scheme MRS-SD-BL is better than others, but it does not outperform M-Nearest scheme.

Figure 6a illustrates the average throughput comparison of existing schemes MRS-ST and MRS-SD (our previous work [10]), HRS [12] and generic schemes, with the increase in radius of search ring. The average throughput of these schemes with relay having full battery power are compared against throughput of the schemes with relay having different battery power levels.

The key observation in Figure 6a is that choosing a relay node nearest to midpoint results in higher throughput among all others, in a network having strong social trust among network users. In addition to that, battery power on a relay device does not significantly affect the average throughput of different relay selection schemes analyzed, except for MRS-ST. The throughput of MRS-ST with varying battery power (the blue dashed line) is reduced for larger value of search radius compared to the scheme with full battery power (the blue line with squares).

Figure 6b illustrates the average throughput comparison of different relay selection schemes with a relay having varying battery power levels. Our proposed schemes MRS-ST-BL and MRS-SD-BL, which takes battery power of devices into consideration for selection of a relay, are compared against existing schemes MRS-ST, MRS-SD, HRS and M-Nearest scheme with a relay having different battery levels. We also analyze the performance of modified version of HRS, HRS-BL, in which relay selection is done considering battery power of devices.

The key observation in Figure 6b is that M-Nearest-WFB (the dashed pink line) has highest throughput among all the schemes analyzed, when users in the network have strong social trust between them. Selecting a relay nearest to midpoint having battery power above battery threshold ($B_{thres}$) reduces throughput of the communication because a relay with longer link lengths is selected if the battery power of a device nearest to the midpoint is less than $B_{thres}$. In this social trust scenario, our schemes perform worse than M-Nearest-WFB. The throughput of our proposed scheme MRS-ST-BL (the blue line with crosses) is greater than that of MRS-ST-WFB (the blue dashed line) for large values of search radius, but less than throughput of M-Nearest-WFB. The performance of our proposed scheme MRS-SD-BL is same as that of MRS-SD-WFB.

In summary, from our analysis we see that average throughput of all schemes decreases when relay has varying battery power levels. Our proposed scheme MRS-ST-BL significantly improves the throughput compared to HRS in all of the analyzed network scenarios. MRS-ST-BL has higher throughput among all others, particularly when social trust among the users in the network are weak. This shows that throughput is improved when the battery power of device is also taken into consideration during relay selection, together with social information of users in the network.

Next, we compare the average number of probes and average transmission power of relay of our proposed schemes with different other schemes. This shows how the limitation in number of probes and average relay power in MRS-ST-BL contribute to enhance throughput of D2D communications.

#### 5.3.2. Comparison of Average Number of Probes of Proposed Schemes with Others

This section presents the comparison of average number of probes of our proposed MRS-ST-BL and MRS-SD-BL schemes with various relay selection schemes. The analysis is done for different social trust scenarios and different node densities. The key result from our analysis is that our proposed schemes probe less number of devices for selection of a relay compared to existing schemes. As a result, our proposed schemes significantly minimize total time spent for probing, which contributes for the enhancement of throughput in D2D communications.

**Figure 7:**
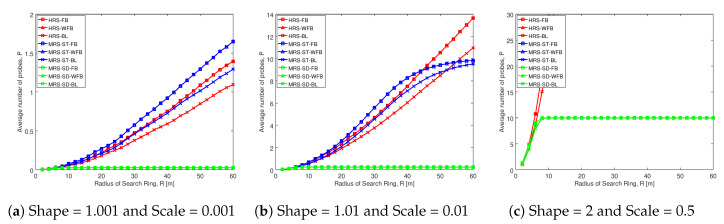
Average probe number comparison of various schemes in different social trust scenarios when network width is 100 m.

Figure 7 illustrates the average number of probes comparison of various schemes for different social trust scenarios in square network having width of 100 m. The average number of probes is on the y-axis and radius of search ring is on the x-axis in each of the graphs. Each of the schemes analyzed are represented by a line.

Figure 7a shows that the average number of probes of MRS-ST-FB (blue line with squares) and MRS-ST-WFB (blue line with triangles) increase with the increase in search radius. From the observation, we can see that MRS-ST-FB and MRS-ST-WFB probe equally with the change in search radius. However, the our proposed scheme MRS-ST-BL (blue line with crosses) has less number of probes compared to MRS-ST-FB and MRS-ST-WFB. This is because MRS-ST-BL only probe those devices having battery level above battery threshold, from the list of devices in circular region that have social trust above social threshold. The reduction in probe number compared to MRS-ST-FB and MRS-ST-WFB contributes to increase throughput. However, the increment is not significant because this is a scenario where social trust among users in the network are very weak. Similar trend is observed when compared between HRS-FB (red line with squares), HRS-WFB (red line with triangles) and HRS-BL (red line with crosses).

Figure fig:Average number of probes of different schemes NW100mb depicts that the average number of probes of MRS-ST-BL (blue line with crosses) is less than that of MRS-ST-FB (blue line with squares) and MRS-ST-WFB (blue line with triangles). The difference in number of probes initially increases with the increase in search radius. This is because, with the increase in search radius more devices (within probe limit) are probed in MRS-ST-FB and MRS-ST-WFB. However, in case of MRS-ST-BL, it filters out the devices with battery less than battery threshold. When the search radius is further increased, the blue lines tend to converse as more devices are probed (up to probe limit) for MRS-ST-FB, MRS-ST-WFB and MRS-ST-BL.

As there is no probe limit in HRS-FB, HRS-WFB and HRS-FB, these schemes probe more devices with the increase in search radius. MRS-SD-FB, MRS-SD-WFB and MRS-SD-BL probe least among all others because these schemes filter at most *l* (probe limit) devices which are nearest to the midpoint before filtering devices that have social trust above social threshold. This is a scenario when social trust among users in the network are weak. Therefore, less number of devices are probed, which helps to increase the throughput of D2D communications.

Figure fig:Average number of probes of different schemes NW100mc shows that the average number of probes for MRS-ST-FB, MRS-ST-WFB, MRS-ST-BL, MRS-SD-FB, MRS-SD-WFB and MRS-SD-BL are indifferent and have number of probes equal to probe limit, with the increase in search radius. This is because the social trust among the users in the network are high, all having social trust above social threshold. However, HRS-FB and HRS-WFB probe more devices compared to HRS-BL. The number of probes for HRS-FB, HRS-WFB and HRS-BL are significantly higher compared to our proposed schemes. Therefore, our proposed schemes have significantly higher throughput compared to HRS-FB, HRS-WFB and HRS-BL.

**Figure A5:**
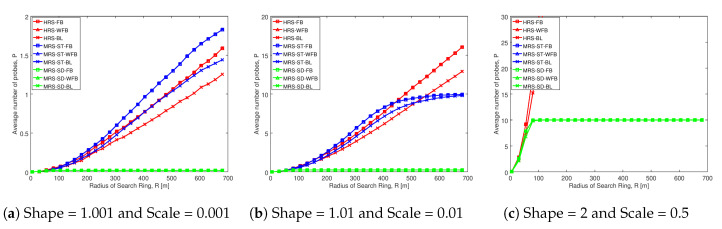
Average probe number comparison of various schemes in different social trust scenarios when network width is 1000 m.

From the comparison of Figure 7a–c, we can also see that the number of probes for all the schemes increase with the increase in social trust among users in the network (figures from left to right). Figure A5 illustrates similar trend in change of probe number with the increase in search radius for different social trust scenarios, when node density in network is low.

From the above analysis, we can see that taking battery level of devices into consideration while selecting a relay, filters out socially trusted devices but with low battery power. It reduces the number of devices to be probed. This helps to select only those devices with high social trust and battery level above battery threshold, as candidate relay devices for D2D communications. As a result, our proposed scheme MRS-ST-BL enhances throughput of D2D communications.

#### 5.3.3. Comparison of Average Relay Power of Proposed Scheme MRS-ST-BL with MRS-ST-FB and MRS-ST-WFB

This section presents the comparison of average power of relay device used in our proposed scheme MRS-ST-BL with MRS-ST-FB and MRS-ST-WFB. The average power of relay selected in HRS (HRS-FB, HRS-WFB, HRS-BL) schemes are not compared because we can see from results obtained in Section 5.3.1 that the performance HRS (HRS-FB, HRS-WFB, HRS-BL) schemes are significantly lower than that of MRS-ST-BL in all the scenarios considered. We show that our proposed scheme MRS-ST-BL enhances the throughput by selecting relay with higher transmission power compared to MRS-ST-WFB, and is the focus of this section. The analysis is done for different social trust scenarios and different node densities. The main result from our analysis is that our proposed scheme MRS-ST-BL selects relay with higher transmission power compared to that of HRS-ST-WFB. As a result, our proposed scheme MRS-ST-BL significantly enhances the throughput of D2D communications.

**Figure 8:**
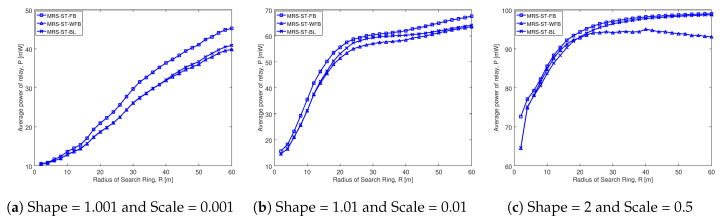
Average transmission power comparison of various schemes in different social trust scenarios when network width is 100 m.

Figure 8 shows the average transmission power of relay of MRS-ST-FB, MRS-ST-WFB and MRS-ST-BL for different social trust scenarios in square network having width of 100 m. The average transmission power of a relay is on the y-axis and radius of search ring is on the x-axis in each of the graphs. Each of the schemes analyzed are represented by a line. The blue line with squares represents transmission power of relay for MRS-ST-FB scheme. This scheme is same as that proposed in Reference [10] and has devices with full battery level in the network. The blue line with triangles represents transmission power of relay for MRS-ST-WFB scheme. This scheme is also same that proposed in Reference [10]. The only difference is that devices in the network have variable battery levels and transmit at lowest power possible when their battery level is lower than battery threshold. The blue line with crosses represents transmission power of MRS-ST-BL, a relay selection scheme proposed in this paper.

Figure 8a shows that the average transmission power of a relay increases with the increase in search radius for all the schemes. The transmission power of relay in MRS-ST-WFB (blue line with triangles) is lower than that of MRS-ST-FB (blue line with squares), as expected. As our proposed scheme MRS-ST-BL (blue line with crosses) also takes battery level into consideration while selecting a relay, it selects a relay with high transmission power compared to MRS-ST-WFB. The selection of higher transmission power can be observed only for large values of search radius. This is because the scenario considered here has very weak social trust among users in the network. Therefore, most of the devices do not transmit at high power even though their battery level is above the threshold value.

Figure 8b shows that the transmission power of all the schemes increases with the increase in search radius. The transmission power of MRS-ST-BL gradually increases and becomes higher than that of MRS-ST-WFB, which can be observed when the search radius is greater than 20 m. However, for larger search radius, the difference in transmission power of MRS-ST-BL and MRS-ST-WFB becomes small.

Figure 8c depcits that the transmission power of relay increases with the increase in search radius for MRS-ST-FB and our proposed scheme MRS-ST-BL. Our proposed scheme MRS-ST-FB selects relay with high transmission power by probing devices having high social trust as well as device battery level above battery threshold. The importance of the battery threshold is quite evident in the transmission power of a relay in MRS-ST-WBL. Interestingly, the transmission power of relay in MRS-ST-WFB is not improved for larger search radius. MRS-ST-WFB filters devices based on social trust, not based on battery level. Therefore, when a device having high social trust but with low battery power is selected as a relay, it transmits at a low power. As a result, throughput of D2D communication is reduced compared to our proposed scheme MRS-ST-BL.

**Figure A6:**
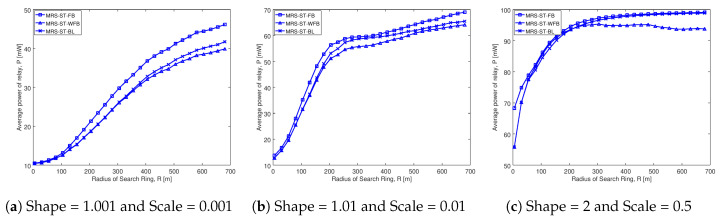
Average transmission power comparison of various schemes in different social trust scenarios when network width is 1000 m.

From the comparison of Figure 8a–c, we can see that the transmission power of relay increases for all the schemes with the increase in social trust among users in the network (figures from left to right). Figure A6 illustrates similar trend in change of relay transmission power with the increase in search radius for different social trust scenarios, when node density in network is low.

From the above analysis, we can see that our proposed scheme MRS-ST-BL selects relays with higher transmission power compared to MRS-ST-WFB. This increment in transmission power of relay contributed to improve average throughput of MRS-ST-BL in Figure 3b, Figure A1b, Figure 4b, Figure A2b, Figure 6b and Figure A4b.

#### 5.3.4. Impact of Battery Threshold on Throughput of Proposed Scheme MRS-ST-BL

**Figure 9:**
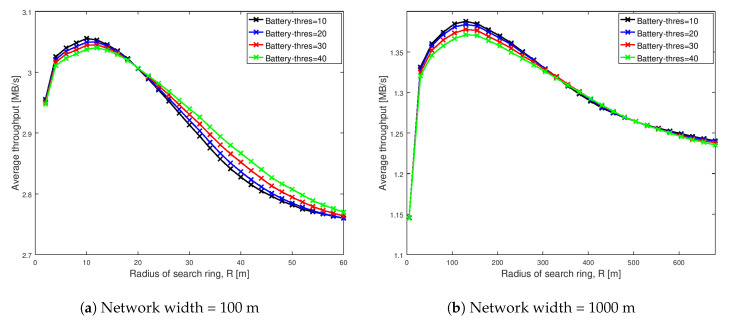
Average throughput of our proposed scheme MRS-ST-BL at different battery threshold when Shape = 1.01 and Scale = 0.01.

This section presents the impact of change in battery threshold ($B_{thres}$) on average throughput of the proposed scheme MRS-ST-BL. Figure 9 illustrates the change in average throughput of MRS-ST-BL as the search radius increases for network widths of 100 m and 1000 m. The key observation from the figure is that the maximum throughput attained by our proposed scheme MRS-ST-BL reduces with the increase in battery threshold.

Figure 9a shows the impact analysis of battery threshold on performance of our proposed scheme MRS-ST-BL as the search radius increases in a network having width of 100 m. The black crossed line represents the battery threshold of 10%, blue crossed line represents that of 20%, the red crossed line represents that of 30% and the green crossed line represents that of 40%. The main result from this figure is that the maximum throughput attained by our proposed scheme MRS-ST-BL reduces with the increase in battery threshold. The throughput initially increases with the increase in search radius for all battery threshold ($B_{thres}$) analyzed. With the further increase in search radius, the throughput gradually decreases. Interestingly, the maximum throughput attained by the proposed MRS-ST-BL for each $B_{thres}$ is different. For $B_{thres}$ = 10, the black crossed line, the throughput attained is maximum among the others. However, for large values of search radius, its throughput is lower than others. When the value of $B_{thres}$ is high, the maximum throughput attained by MRS-ST-BL is reduced. However, for large search radius, its throughput is greater compared to the scheme when $B_{thres}$ has less value.

Figure 9b shows the impact analysis of battery power threshold on performance of our proposed scheme MRS-ST-BL as the search radius increases in a network having width of 1000 m. The main result from this graph is the difference in maximum throughput attained at various $B_{thres}$ is larger compared to that in Figure 9a. In addition to that, the throughput of MRS-ST-BL at various $B_{thres}$ are indifferent for large values of search radius as compared to that in Figure 9a.

This shows that the performance of proposed MRS-ST-BL also depends upon the battery threshold. The proper adjustment of the battery threshold in our proposed scheme MRS-ST-BL results in increased throughput of D2D communications.

## 6. Conclusions

The devices in a network may have different battery power levels. When transmission power of a device also depends upon its battery power, selection of a relay for D2D communications becomes challenging. In this paper, we have designed midpoint relay selection schemes which selects socially trusted relay that is located around the midpoint of the distance between the source and the destination. Our relay selection designs incorporate battery power level of devices together with social trust information of users. A relay is selected for D2D communications only when the throughput of D2D communications via a relay is greater than that of direct D2D communications.

When the devices in a network have different battery power levels, the performance of all of the schemes analyzed decreases. However, with the use of proposed relay selection selection scheme in this paper, the performance can be significantly improved. We compared the performance of existing relay selection schemes including HRS, MRS-ST, MRS-SD and other generic schemes, when relay devices have different power levels. The results showed that the performance of the schemes are not as good as originally claimed. Then, we compared the average throughput of our proposed schemes against state-of-the-art schemes including HRS, MRS-ST, MRS-SD and other generic schemes, when devices in the network have different battery power levels. We analyzed the performance for different social trust scenarios and device densities. The proposed relay selection scheme MRS-ST-BL significantly improved throughput of D2D communications in networks with weak social trust among users compared to others. The results show that performance is improved by minimizing number of probes and by selecting a relay with high transmission power. Additionally, we also show that the battery power threshold impacts upon the performance of proposed scheme MRS-ST-BL.

In summary, our proposed relay selection scheme MRS-ST-BL significantly improves throughput of D2D communications compared to other schemes when relay devices have different battery power levels, particularly in networks that have weak social trust among users.

## Figures and Tables

**Table 1:** Simulation setup for analysis of MRS-RP.

Simulation Parameters	
Simulation software	Octave
Mobility model	Random waypoint
Number of nodes, $n_{\mathrm{total}}$	1000
Network width, *W*	100 and 1000 m
Speed of nodes, *S*	0–2 m/s
Maximum pause time	5 s
Simulation period	1000 s
Channel bandwidth, *B*	1 MHz
Pathloss exponent, θ	4
Noise power, *N*	−114 dBm
Social trust, β	0–1
Social threshold, $S_{thres}$	0.3
Battery threshold, $B_{thres}$	10, 20, 30, 40
Probe limit, *l*	10
Shape, α	1.001, 1.01, 1.1, 2
Scale, *L*	0.001, 0.01, 0.1, 0.2, 0.5
Upper limit, *H*	1
Transmission power, $P_{s,d}$	20 dBm
Max. allowable tx power, $P_{s,j}$	20 dBm
Time slot duration, *t*	1 s
Probe duration, τ	0.01 s
Search radius, *R*	Upto 700 m

## Appendix A

### Appendix A.1. Impact of Different Battery Levels of Relays on Throughput in Low Node Density Network

This section presents additional analysis comparing average throughput of our proposed MRS-ST-BL and MRS-SD-BL schemes with various relay selection schemes, when network width is 1000 m. We analyzed average throughput of each schemes, where search radius ranges from 5 m upto 700 m. The simulation data are recorded at an interval of 25 m.

Figure A1, Figure A2, Figure A3 and Figure A4 show the throughput comparison at different social trust scenarios for a low node density network.

Figure A1 depicts a very weak social trust scenario (Shape = 1.001, Scale = 0.001) among users in a network with low node density. The change in throughput with the increase in radius of search ring for all relay selection schemes are similar to those in Figure 3.

Figure A2 shows the same scenario as Figure 4, except the density of devices are less in Figure A2. The throughput response is about the same as in Figure 4.

Figure A3 depicts average throughput comparison of different schemes for a social trust scenario (Shape = 1.1, Scale = 0.1) among users, which is stronger than in Figure A2, in a network with low node density. The trend of change in throughput of all the schemes analyzed are similar to those in Figure A2.

Figure A4 depicts average throughput comparison of different schemes for a strong social trust scenario (Shape = 2, Scale = 0.5) among users in a network with low node density. The change in throughput with the increase in search radius for all the schemes analyzed are not different to those in Figure 6.

In summary, our proposed schemes MRS-ST-BL and MRS-SD-BL provide higher throughput compared to existing art-of-the-state schemes in a low node density network having devices with varying battery levels.

### Appendix A.2. Average Number of Probes Comparison of Different Schemes in Low Node Density Network

This section presents additional analysis comparing average number of probes of our proposed MRS-ST-BL and MRS-SD-BL schemes with various relay selection schemes, when network width is 1000 m. The analysis is done for different social trust scenarios. We analyzed average number of probes of each schemes where search radius ranges from 5 m upto 700 m and simulation data are recorded at an interval of 25 m.

Figure A5 shows that the change in average number of probes with the increase in search radius for all the schemes analyzed are similar to those in Figure 7. This result shows that our proposed schemes perform better by probing comparatively lower than other schemes in a network having low node density.

### Appendix A.3. Average Power of Relay Comparison of MRS-ST-FB, MRS-ST-WFB and MRS-ST-BL in Low Node Density Network

This section presents additional analysis comparing the average relay power of our proposed scheme MRS-ST-BL with MRS-ST-FB and MRS-ST-WBF, when network width is 1000 m. The analysis is done for different social trust scenarios. We analyzed average relay transmission power of each schemes where search radius ranges from 5 m upto 700 m and simulation data are recorded at an interval of 25 m.

Figure A6 shows that the change in average transmission power of relay with the increase in search radius for all the schemes analyzed are similar to those in Figure 8.

From the results, we can conclude that our proposed scheme MRS-ST-BL performs better by selecting relay with higher transmission power compared to MRS-ST-WBL, when node density in the network is low.
